# Next-Generation Autoantibody Testing by Combination of Screening and Confirmation—the CytoBead® Technology

**DOI:** 10.1007/s12016-016-8574-3

**Published:** 2016-07-02

**Authors:** Mandy Sowa, Rico Hiemann, Peter Schierack, Dirk Reinhold, Karsten Conrad, Dirk Roggenbuck

**Affiliations:** 1 GA Generic Assays GmbH, Dahlewitz, Berlin, Germany; 2 Institute of Biotechnology, Faculty of Environment and Natural Sciences, Brandenburg University of Technology Cottbus-Senftenberg, Senftenberg, Germany; 3 Institute of Molecular and Clinical Immunology, Otto-von-Guericke-University Magdeburg, Magdeburg, Germany; 4 Institute of Immunology, Medical Faculty, Technical University Dresden, Dresden, Germany

**Keywords:** Second-generation autoantibody testing, Indirect immunofluorescence, Digital fluorescence, Autoimmune disease, Multiplex diagnostics

## Abstract

Occurrence of autoantibodies (autoAbs) is a hallmark of autoimmune diseases, and the analysis thereof is an essential part in the diagnosis of organ-specific autoimmune and systemic autoimmune rheumatic diseases (SARD), especially connective tissue diseases (CTDs). Due to the appearance of autoAb profiles in SARD patients and the complexity of the corresponding serological diagnosis, different diagnostic strategies have been suggested for appropriate autoAb testing. Thus, evolving assay techniques and the continuous discovery of novel autoantigens have greatly influenced the development of these strategies. Antinuclear antibody (ANA) analysis by indirect immunofluorescence (IIF) on tissue and later cellular substrates was one of the first tests introduced into clinical routine and is still an indispensable tool for CTD serology. Thus, screening for ANA by IIF is recommended to be followed by confirmatory testing of positive findings employing different assay techniques. Given the continuous growth in the demand for autoAb testing, IIF has been challenged as the standard method for ANA and other autoAb analyses due to lacking automation, standardization, modern data management, and human bias in IIF pattern interpretation. To address these limitations of autoAb testing, the CytoBead® technique has been introduced recently which enables automated interpretation of cell-based IIF and quantitative autoAb multiplexing by addressable microbead immunoassays in one reaction environment. Thus, autoAb screening and confirmatory testing can be combined for the first time. The present review discusses the history of autoAb assay techniques in this context and gives an overview and outlook of the recent progress in emerging technologies.

## Autoantibodies as Diagnostic Markers

### Connective Tissue Disease-Specific Autoantibodies

**Fig. 1:**
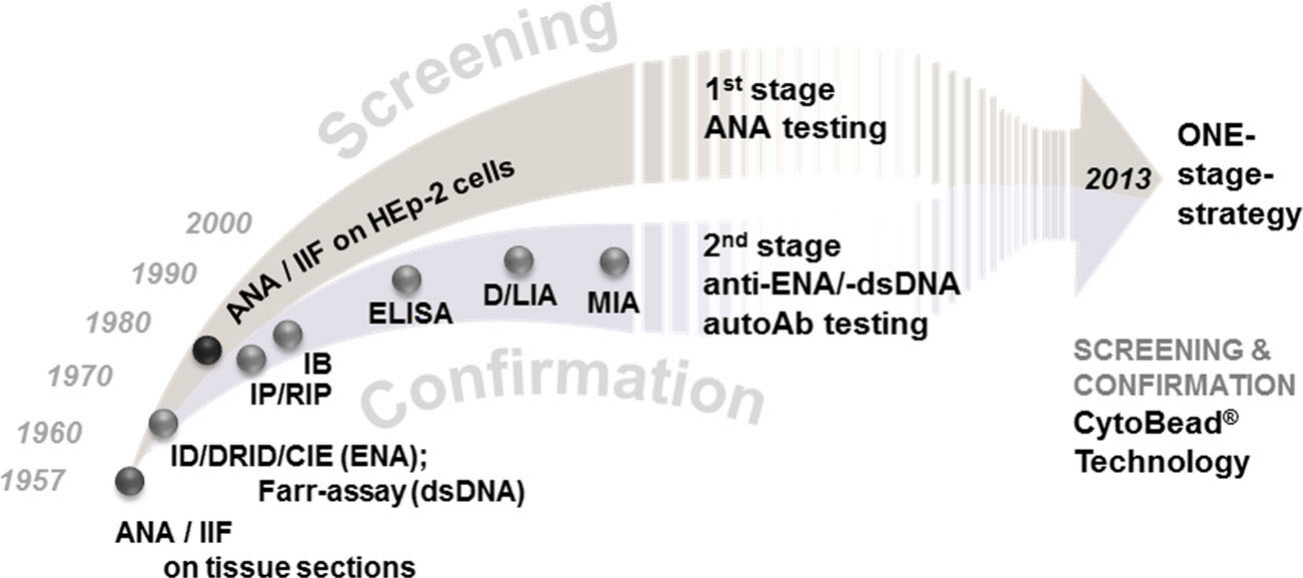
Evolving autoantibody (autoAb) testing and strategies for the serological diagnosis of systemic autoimmune rheumatic diseases. *ANA* antinuclear antibody, *autoAb* autoantibody, *CIE* counterimmunoelectrophoresis, *D/LIA* dot/line immunoassay, *ELISA* enzyme-linked immunosorbent assay, *ENA* extractable nuclear antigen, *IB* immunoblot/westernblot, *ID/DRID* immunodiffusion/double radial immunodiffusion, *IIF* indirect immunofluorescence, *IP* immunoprecipitation, *MIA* microbead immunoassay, *RIP* radioimmunoprecipitation

The loss of immune tolerance characteristic for connective tissue diseases (CTDs) such as systemic lupus erythematosus (SLE), systemic sclerosis (SSc), poly/dermatomyositis (PM/DM), Sjögren’s syndrome (SjS), and mixed connective tissue disease (MCTD) brings about the generation of various nonorgan-specific autoantibodies (autoAbs) [1–3]. Although the triggering factors for the occurrence of autoAbs and their role in the pathogenesis of CTD are still not entirely understood, autoAbs are widely used as diagnostic markers in clinical routine nowadays [4, 5]. The L.E. cell phenomenon described by Hargraves in the late 1940 in patients suffering from SLE proved to be a result of autoAb binding to nuclear material of polymorphs and marked the beginning of a rapidly evolving autoAb era in clinical diagnostics [6]. Indirect immunofluorescence (IIF) was the first assay technique employed to reveal autoAbs in patients with CTD [7]. The groundbreaking works of Holborow and Friou et al. led to the discovery of so-called antinuclear antibodies (ANAs) as marker autoAbs of CTD like SLE [8, 9]. In the following years, clinicians made tremendous efforts to understand the clinical significance of autoAbs and their potential use for the serological diagnosis of CTD and beyond [10]. This process was greatly driven by novel emerging assay techniques used for autoAb testing and their respective assay performance characteristics (Fig. 1; Table 1). The ensuing discourse has led to the definition of various diagnostic strategies for the serological diagnosis of autoimmune disorders and continues to date. Of note, ANA detected by IIF was included into the diagnostic criteria of SLE and autoimmune hepatitis (AIH) later [11–13]. In this context, the discovery of autoAbs to extractable nuclear antigens (ENAs) apart from autoAbs to dsDNA or histones in the search for disease-specific autoAbs provides an intriguing example for the change in the understanding of the clinical meaning of autoAbs as diagnostic markers [14–16]. Thus, the seminal paper of E.M. Tan and H.G. Kunkel on the identification of Sm as an autoantigenic target of SLE and the use of double radial immunodiffusion (DRID; Ouchterlony technique) for its detection ushered in a new era in autoAb diagnostics and its clinical application [17]. Although ANA turned out to be a sensitive marker for SARD as a whole disease group, its specificity for distinct SARD entities was not satisfactory despite being defined as a diagnostic marker for SLE [11]. Thus, the clinical need for more specific “ANA” was met by the pioneering work of H.G. Kunkel, E.M. Tan, and others discovering more and more novel autoAbs to ENA with clinical significance [14, 18]. However, not all ENAs identified as targets for CTD-specific autoAbs could be isolated by the saline extraction technique reported previously and should not be termed ENA [19]. Furthermore, apart from autoAbs recognizing nuclear autoantigens, anticytoplasmic autoAbs (ACyA) have been introduced into the autoAb panel for SARD serology [20]. Thus, the anti-SjS antigen A (SS-A) autoAbs also termed Ro have been shown to interact with its respective target in the cytoplasm [21]. As a fact, the progress in proteomics enabled the identification of cytoplasmic autoantigenic targets interacting with for instance myositis-specific autoAbs like anti-histidyl tRNAse autoAbs (Jo-1) or SLE specific autoAb against ribosomal proteins [22–24]. Obviously, this created confusion among clinical and laboratory experts and called for clarification. In terms of ANA testing, the introduction of human epidermoid laryngeal carcinoma (HEp-2) cells as improved autoantigenic substrate in IIF has encouraged the reporting of CTD-specific cytoplasmic patterns over the years [2]. This contradiction in terminology was addressed by a recent consensus recommending the use of anticellular antibodies instead of ANA [4]. Notwithstanding, the use of ANA and ENA is well established particularly among clinicians and it remains to be seen how this issue will be solved adequately in the years to come [25]. In summary, autoAb testing is an integral part in the serological diagnosis of CTD and may also assist in the prognosis, subclassification, as well as monitoring of disease activity [4, 10, 26–29].

**Table 1:** Autoantibody (AAB) detection methods in routine diagnostics of systemic rheumatic diseases

Method	Principle	Advantages	Disadvantages	Application
Chip technique (Spot immunoassay) [142, 168, 172–175]	autoAb binding to purified native or recombinant proteins immobilized as a spot on an adsorbent membrane, measurement: see ELISA	•More autoAb per test detectable compared to DIA/LIA •Very low amount of autoantigens needed	•Optimal epitope presentation for each autoantigen difficult to achieve •Possible interferences (see ELISA) may lead to false positive reactions	Multiparametric determination of autoAb
*Crithidia luciliae* Immunofluorescence Test [108, 110–113, 191]	In situ autoAb binding to kinetoplast DNA of *Crithidia luciliae*, visualization of autoAb binding by fluorescence-labeled anti-human IgG	High diagnostic specificity for SLE	•Low diagnostic sensitivity for SLE •Semiquantitative analyses only	Determination of dsDNA autoAb in suspicion of SLE or in sera with homogeneous ANA pattern
DIA/LIA [90, 116, 140, 151–159]	autoAb binding to purified native or recombinant proteins immobilized as dot or line on an adsorbent membrane, measurement: see ELISA	•Allows the specific detection of numerous autoAb per test including vary rare autoAb •Low amounts of autoantigens needed	•Qualitative or semi-quantitative analyses only •Possible interferences (see ELISA) may lead to false positive reactions	Multiparametric determination of autoAb (e.g., myositis or SSc specific autoAb)
Double radial immunodiffusion (Ouchterlony technique) [14, 16, 17, 21, 31, 90, 113]	Precipitation of the autoAb with the corresponding soluble autoantigen in gel after radial immunodiffusion; determination of autoAb specificity by reference antibodies	High diagnostic specificity for CTD	•Low diagnostic sensitivity for CTD •Time-consuming (24–48 h)	Screening for autoAb against ENA in serum of patients with suspected CTD
ELISA [3, 22, 32, 37, 53, 62, 71, 80, 95, 101, 120–122, 184]	autoAb binding to solid-phase (multiwell plate) immobilized autoantigen, measurement of autoAb interaction by enzyme-labeled anti-human IgG (or IgA, or IgM): colorimetry by substrate conversion with proportional behaviour to the strength of immune reaction	•Versatile and sensitive analytical technique •Good quantification •Good automation •Quick and cost-effective •Differentiation of immunoglobulin classes possible	Interferences may lead to false positive reactions (cross-reactive autoAb, matrix effects, endogenic proteins, nonspecific binding, autoAb against blocking proteins)	Specific determination of autoAb (highly purified native or recombinant autoantigens are required)
Farr radioimmunoassay [7–9, 55, 57, 67, 96, 106, 205]	Precipitation of anti-dsDNA/DNA complexes; Measurement of the quantity of dsDNA autoAb by using radioactively labeled dsDNA	•High diagnostic specificity for SLE •Superior for monitoring lupus disease activity compared to ELISA	•Requires radioactive material •Higher effort compared to ELISA	Specific detection and quantification of dsDNA autoAb
IIF on HEp-2 cells [7–9, 55, 57, 67, 96, 106, 205]	In situ autoAb binding to antigens of HEp-2 cells, visualization of autoAb binding by fluorescence molecule labeled anti-human IgG	•High sensitive detection of most clinically relevant nonorgan-specific autoAb •Optimal combination of immunoassays for further evaluation of specific autoAb taking into account IIF pattern and suspected diagnosis •Detection of diagnostically relevant autoAb without further need of specific immunoassays (e.g., centromere autoAb) •Assessment of autoAb only detectable by this method since the autoantigenic targets have not been identified or commercial assays are not available yet	•Subjective assessment •Reliable results require qualified and experienced lab personnel •High intralaboratory and interlaboratory variance → Automatic image recognition and interpretation improves and standardizes results	autoAb screening in sera of patients suspected of having SARD or autoimmune liver disease
Microparticle based immunoassays [102, 138, 139, 142, 164, 170–172, 176–179, 206–208]	autoAb bind to antigens immobilized on beads; measurement by flow cytometry (suspension bead assay) or optical microscope (planar bead assay)	•More autoAb per test detectable compared to DIA/LIA •Very low amount of autoantigens needed •Better epitope presentation for each autoantigen compared to spot assay •Combination with IIF possible (CytoBead® assay)	•Possible interferences (see ELISA) may lead to false-positive reactions	Multiparametric determination of autoAb
Passive agglutination (Latex test: RF) [216]	Binding of RF to human IgG bound on the surface of biologically inactive latex particles leads to visible agglutination of the particles	•Easy to perform •No need of instruments •High sensitivity	•Qualitative or semi-quantitative analyses only •False-positive reaction if reaction time is surpassed •Intensity of agglutination does not correlate with RF titer •Low specificity	Screening for RF (only rarely used in routine diagnostic since introduction of CCP autoAb)
Passive hemagglutination (Waaler-Rose test: RF) [217]	Binding of soluble autoantigens coated on red blood cells leads to visible erythrocyte agglutination	•Easy to perform •No need of instruments	•Qualitative or semi-quantitative analyses only •Subjective assessment	Not used anymore in routine diagnostics (in the past used for detection of RF, dsDNA, and Sm/RNP autoAbs)
Radioimmuno-precipitation assay [124, 125, 129]	autoAb binding to autoantigens of radiolabelled cell extracts; analyses of bound antigens by autoradiography after gel electrophoresis of the immunoprecipitates	Allows the detection of numerous autoAb without purification of autoantigens	•Requires radioactive material •Higher effort	Not used in routine practice; may be used for assay comparison and to search for novel autoAb (specialized labs only)
Westernblot (Immunoblot) [81, 89, 113]	autoAb binding to electrophoretically separated proteins transferred to adsorbent membrane, measurement: see ELISA	Allows the detection of numerous autoAb without purification of autoantigens	•False-negative results due to destroyed (denaturation of proteins during electrophoresis) or masked epitopes •False-positive results due to comigrated proteins	Not used anymore in routine diagnostics; may be used to search for novel autoAb
Nephelometry [218]	The amount of antigen/antibody complexes were measured by light scatter	•Easy to perform •Less-time consuming •Greater precision compared to latex test (see passive agglutination)	•No discrimination between isotypes •Lower diagnostic sensitivity compared to ELISA	Quantification of RF

*ANA* antinuclear antibody, *autoAb* autoantibody, *CCP* cyclic citrulinated peptide, *CTD* connected tissue disease,* DIA/LIA* dot/line immunoassay, *ELISA *enzyme-linked immunosorbent assay, *ENA* extractable nuclear antigen, *IIF* indirect immunofluorescence, *RF* rheumatoid factor, *SARD* systemic autoimmune disease, *SLE* systemic lupus erythematosus, *SSc* systemic sclerosis

As mentioned earlier, not only the discovery of novel SARD-specific autoAbs has challenged the diagnostic skills of clinicians but the introduction of novel assay techniques with differing assay performance, too [30]. Thus, the change from immunodiffusion-based detection techniques like DRID or counterimmunoelectrophoresis (CIE) detecting precipitating autoAbs to enzyme-linked immunosorbent assay (ELISA) regarding the analysis of autoAbs to Sm or SS-A called the specificity of these distinct markers suddenly into question [31–33]. The solid-phase ELISA brought about a significantly elevated sensitivity which in turn is related to a diminished diagnostic specificity [34]. Furthermore, with the better understanding of the chemical structure of for instance the small nuclear ribonucleoprotein (snRNP) complex representing the Sm autoantigen, six different protein structures (B, B’, D, E, F, G) were identified as autoantigenic targets with SmD being apparently the most specific one for SLE [35–37]. Alone, these critical aspects require a comprehensive knowledge on the interpretation of assay characteristics by clinicians which were not always conveyed by laboratorians adequately [1, 3]. The badly needed comprehension of pretest and posttest probabilities of presence of disease and its relation to the diagnostic performance of autoAb analysis such as ANA testing appears not satisfactorily developed in clinicians [19, 38, 39]. Thus, novel diagnostic strategies translating the progress in autoAb testing proved difficult to get in line with established diagnostic pathways [27, 40, 41]. The recent attempt to substitute ANA IIF testing as screening assay within the two-tier strategy by novel multiplex techniques failed or met with great resistance among rheumatologists [4, 42, 43]. Consequently, the two-stage strategy recommending ANA testing by IIF as screening and appropriate confirmation of ANA positives by a different analysis was confirmed by expert consensus for CTD serology recently [4].

### Autoimmune Vasculitis-Specific Autoantibodies

Of note, like revealed for the L.E. phenomenon in patients with SLE, patients suffering from autoimmune vasculitides demonstrate loss of tolerance to polymorphs, too [44]. In contrast, the occurring autoAbs recognize specific neutrophil cytoplasmic and not nonspecific nuclear components and were described first in association with glomerulonephritis in 1982 by Davies et al. [45]. Van de Woude’s group reported so-called antineutrophil cytoplasmic antibodies (ANCAs) to be associated with granulomatosis with polyangiitis (GPA, formerly Wegener’s granulomatosis) shortly later and, consequently, the term ANCA-associated vasculitides (AAV) was coined [44, 46, 47]. Thus, this group of autoimmune vascular disorders comprises GPA, microscopic polyangiitis (MPA), and eosinophilic granulomatosis with polyangiitis (EGPA, formerly Churg-Strauss syndrome) [48, 49]. Their leading clinical characteristics are microvascular inflammation, tissue necrosis, and the appearance of ANCAs [50].

Interestingly, similar to ANA testing, IIF was the first method to be used for the detection of ANCA revealing two major patterns, cytoplasmic (cANCA) and perinuclear ANCA (pANCA) [45, 51]. Not surprisingly, the respective main autoantigenic neutrophilic targets, proteinase 3 (PR3), and myeloperoxidase (MPO) were discovered shortly afterward [52, 53]. Consequently, a two-stage strategy for ANCA testing highlighting IIF as a standard method is recommended by international consensus for the serology of AAV, too [54]. Indeed, the unsurpassed high sensitivity of autoAb analysis employing cellular substrates by IIF renders this method an ideal tool for the screening stage followed by confirmatory testing with different immunological assay technologies [47]. However, similar to ANA IIF reading, interpretation of ANCA patterns is rather time consuming due to lack of automation and skilled laboratory experts [55]. Thus, IIF is in general highly subjective what renders appropriate standardization difficult [56, 57]. Therefore, attempts to replace IIF by novel techniques based on solid-phase immunoassays (e.g., ELISA, dot/line immunoassay, addressable bead/microarray assays) for ANCA as well as ANA analyses are increasing currently [58–62]. Indeed, in contrast to IIF, these assay techniques can be automated and proved to be more cost-efficient in the modern laboratory environment characterized by a rising diagnostic demand due to the growing clinical impact of autoimmune diseases. However, worrying rates of false-negative findings have been reported for these techniques in terms of ANA as well as ANCA testing [42]. Of note, this fact also appears to be relevant for organ-specific autoimmune disorders like celiac disease (CD).

### Celiac Disease-Specific (Auto)Antibodies

Celiac disease, a gluten-related and immune-mediated small intestinal disease, is one of the few autoimmune disorders which the triggering factor was identified for [63]. Indeed, gliadin peptides deamidated by tissue transglutaminase type 2 (TG2) were shown as gluten-related T-cell epitopes triggering chronic inflammatory intestinal lesions and leading to villous atrophy and hyperplasia of the crypts [64].

Like for CTD and AAV, serology is paramount for the diagnosis of CD encompassing the detection of (auto)Abs to endomysium (EmA), deamidated gliadin peptides (DGP), and TG2 of the IgA isotype [65]. As a fact, due to the excellent assay performance of EmA testing by IIF, this particular autoAb is still considered the reference standard for CD-specific (auto)Abs [65–67]. However, similar to ANA and ANCA testing by IIF, EmA IIF analysis was questioned more and more because it may be subject to interobserver as well as substrate-related variability and is difficult to automate [68]. Obviously, testing of anti-TG2 autoAbs by immunometric solid-phase assays was favored instead [69–72].

In summary, IIF as one of the first techniques employed for autoAb testing in various autoimmune disease diagnostics appears to keep its appeal with laboratorians and clinicians despite several shortcomings [73, 74]. The integration of IIF as screening or standard method for autoAb analysis into two-stage or multiplex strategies was necessary as yet, but creates cost constraints for health care systems already burdened with spiraling costs. This calls for innovative solutions to meet the growing demand for autoAb testing in clinical routine.

## Evolving Assay Techniques for Autoantibody Testing

### Single Tests for autoAb Analysis

The introduction of fluorescent dyes and the development of immunochemical methods for the labeling of antibodies on the one hand and fluorescence microscopy on the other hand paved the way for IIF as powerful tool for autoAb analysis in the 1950s [75]. Thus, the detection of ANA by IIF employing first rodent liver tissue and later HEp-2 cells as autoantigenic substrate marks the beginning of autoAb detection in the serological diagnosis of CTD [7, 9, 76, 77]. However, it turned out soon that the clinical need for disease-specific autoAbs was not appropriately addressed by ANA testing alone. The search for more specific autoAbs led to the introduction of immunodiffusion techniques which enabled the discovery of disease-specific autoAbs like the Sm autoAb in patients suffering from SLE [16, 17, 21]. In particular, DID employing thymic extracts was used and clinicians learnt to appreciate the high specificity of this new parameter for CTD serology. Not surprisingly, autoAbs to Sm were included along with ANA in the diagnostic criteria for SLE later and are still considered as one of the most specific serological parameters for SLE [11, 14]. However, DRID is a time-consuming technique and, thus, was replaced by CIE enabling a faster and more sensitive detection of precipitating autoAbs later on [31]. Several other important autoAbs to the spliceosomal complex such as autoAbs to U1 ribonucleoprotein (U1-RNP) were identified in the quest for new CTD markers [21]. Anti-U1-RNP was established as a specific serological marker for MCTD and found in patients with SLE as well [78, 79]. The introduction of new assay techniques like radio- (RIA) and enzyme immunoassays as well as radio/immunoprecipitation paved the way for the development of autoAb detection assays with better assay performance [32, 62, 80–86]. In particular, the progress in proteomics and the introduction of the immunoblot technique enabled the purification and identification of the distinct autoantigenic targets [33, 87–89]. It turned out that Sm and U1-RNP consist of several autoantigenic components including U1-RNA with different characteristics regarding their performance as split autoantigens especially in solid-phase ELISAs [79, 90, 91]. Furthermore, the SjS-specific autoantigens SS-A and SS-B form a complex interacting with yRNA [92]. Of note, this confers only to the SS-A 60 kDa unit whereas the 52 kDa SS-A (TRIM21) does not bind to yRNA and is not related to this snRNP complex [93, 94]. This raised the question of the best composition of these targets for the detection of the distinct autoAbs or the use of the target subcomponent with the best assay performance [90]. In terms of U1-RNP consisting of components A, C, and a 68 kDa polypeptide, it was found that at least two of these three should be used as solid-phase antigens to set up an appropriate ELISA for the detection of autoAbs to U1-RNP [19]. In contrast, SmD of the Sm complex with its six subcomponents mentioned earlier appeared to be the most specific and sensitive autoantigenic target in ELISA for the serology of SLE [14].

In general, the introduction of solid-phase assays like ELISA was accompanied by four major aspects changing the understanding of autoAb testing for CTD diagnostics: (i) a better usability as assay platform, (ii) an increasing sensitivity compared with immunodiffusion techniques, (iii) the different assay performance of autoAbs recognizing conformational or nonconformational, linear epitopes, and (iv) the introduction of reference sera for standardized diagnostics. This was an essential step toward standardization and automation of autoAb testing addressing the growing demand thereof due to the inclusion of autoAb testing into diagnostic or classification criteria of more and more autoimmune diseases and changed the autoimmune laboratory environment dramatically [80, 95]. Consequently, assay techniques like IIF, which have been prone to subjectivity and difficult to automate until recently, were subjected to a rising pressure to be substituted [73, 96, 97]. In this context, several researchers were tempted by the advantages of the ELISA technique and in particular its higher sensitivity to develop assays employing cellular extracts of MOLT4 or HEp-2 cells [98–101]. Furthermore, the elevated sensitivity of particularly anti-SS-A ELISAs revealed false-negative ANA sera of patients suffering from CTD [102–105]. Indeed, this seems to be the only autoantigenic target which is not adequately presented even by HEp-2 cells and can result in false-negative ANA findings by IIF. To overcome this shortcoming of the appreciated IIF technique, genetically modified HEp-2 cells with a higher expression of the SS-A 60 kDA polypeptide were introduced in ANA testing [103, 106].

Of note, the increased sensitivity of ELISA resulted in positive autoAb findings in nondiseased individuals, too, which started an intense discourse on the right method for cutoff determination [80]. Finally, receiver operating characteristics curve analysis was approved for quantitative methods like ELISA as the best approach to do so [39]. Part of the false-positive findings could be assigned to autoAbs occurring before the onset of disease as putative predictive markers thereof [29, 107]. Nonetheless, false-positive findings in ELISA could be a result of autoAbs to less disease-specific nonconformational epitopes [108]. These autoAbs often belong to the natural autoAb repertoire and display a low affinity to its corresponding targets [109]. A very convincing example is the anti-double-stranded DNA (dsDNA) autoAb which was established as diagnostic marker of SLE [110]. Of note, the SLE-specific dsDNA epitope is ill-defined and IIF assays employing kinetoplast dsDNA of *Crithidia luciliae* (CLIFT) with its characteristic epitope structure appear to provide the best specificity for this important disease activity-associated SLE marker [108, 111–113]. The replacement of CLIFT and the Farr RIA measuring mainly high-affinity anti-dsDNA autoAbs due to a high-salt reaction environment by ELISAs detecting autoAbs to both nonconformational and conformational dsDNA epitopes resulted in high numbers of false-positives particularly in patients with infectious diseases [114].

A similar phenomenon was observed when recombinant or synthetic autoantigens were introduced into autoAb testing to overcome the difficulties related to antigen purification and standardization [115, 116]. Not in each case, these nonnative polypeptides could replace the native autoantigenic targets for an appropriate autoAb analysis. Thus, the SmD polypeptide was dependent on the symmetric methylation of arginine to represent the SLE-specific epitope for the sensitive detection of anti-Sm autoAbs [35, 36, 117]. Furthermore, the presence of yRNA for the autoantigenicity of the SS-A/SS-B complex on the one hand and of U1-RNA for the Sm/RNP unit on the other hand was obviously required for the sensitive analysis of the respective autoAbs [118, 119].

Remarkably, specific ANCA testing demonstrated similar difficulties. Like for ANA testing, IIF was introduced as first assay technique on fixed neutrophils [45]. However, the following identification of PR3 and MPO as the main ANCA targets and the subsequent analysis of respective autoAbs by ELISA were hampered by the nonsatisfactory sensitivity of anti-PR3 autoAb tests [120–122]. Indeed, the conformational epitopes on PR3 were difficult to preserve on the solid phases of ELISAs. Recently, the third generation of PR3-ANCA ELISA has been introduced employing anchor molecules during adsorption of PR3 to the solid phase to preserve its confirmation and accessibility of vasculitis-specific epitopes [121, 122]. Other attempts to develop highly sensitive PR3-ANCA ELISAs comprised the use of a mixture of native as well as recombinant PR3 [123].

The close relation between sensitivity and specificity is presumably the reason that direct-ligand RIAs with their excellent sensitivity have not been used widely for the analysis of CTD- or AAV-specific autoAbs. Interestingly, this is in contrast to organ-specific autoimmune entities such as type 1 diabetes (T1D) where RIAs are appreciated hitherto due to their high sensitivity [124, 125]. Of note, IIF on endocrine pancreas had also been the first technique used for autoAb analysis before the corresponding autoantigens were identified [126]. The detection of islet-cell autoAbs by IIF is still in use; however, the impact of conformational epitopes for T1D autoAbs testing in conjunction with the increased sensitivity of RIAs and recently emerging ELISAs with similar assay performances have almost replaced IIF [127].

After the discovery of TG2 as autoantigenic target of EmA for CD serology, a similar development was observed in the serological diagnosis of CD [128]. To obtain a sensitive anti-TG2 autoAb assay, conformational epitopes of TG2 seem to be essential, too [129]. In contrast to T1D autoAb testing, however, the detection of EmA by IIF is still the gold standard [65].

As a fact, the higher disease specificity of autoAbs to conformational epitopes is probably the reason for today’s infrequent use of immunoblot assays for autoAb serology [130]. Obviously, due to the poor presentation of such epitopes on the blot membrane as a result of the denaturing effect of sodium dodecyl sulfate during electrophoresis and the poor standardization of the method due to technical peculiarities, the immunoblot technique has lost its initial appeal for multiplex autoAb testing [89, 131].

Notwithstanding, due to the progress in the identification of ever more autoAbs aiding in diagnosing, predicting and prognosing autoimmune diseases, the search for the most adequate strategy of autoAb testing fulfilling clinical needs and cost constraints has been in the focus of laboratory and clinical experts ever since [5, 59, 132–134]. For instance, more than 100 autoAbs were found in SLE patients alone [135]. This led to the introduction of fully automated random-access instruments employing fluorescence or chemiluminescence as read out for autoAb testing as well as screening [136–139].

Remarkably, a two-stage strategy was recommended for both ANA and ANCA analyses by international consensus recommendations [4, 54]. Thus, IIF is still considered a reliable screening test characterized by a high negative predictive value. Positive IIF findings should be confirmed by specific autoAb testing employing assay techniques with high specificity. For several other autoimmune disorders like for instance CD, IIF is still considered a gold standard [65]. Thus, despite the introduction of assay techniques for the detection of specific autoAb reactivities, there is still a need for testing of autoAbs by various assay techniques.

### Multiplex Assays for autoAb Testing

The rising number of autoAbs requested for the serology of one autoimmune entity as well as the growing demand for autoAb testing in general encouraged the development of multiplex testing [3, 140–142]. Despite the fact that ANA assessment by IIF using HEp-2 cells as autoantigenic substrate is already a multiplex test revealing different patterns according to the autoAbs present in the serum investigated, the analysis of specific autoAbs is hardly achievable [20, 96, 143, 144]. Even for such ready to detect ANA patterns like the centromere one with its more than 40 fluorescent dots spread in nuclei of interphase cells and densely aligned dots in the metaphase cells, several proteins could be recognized by autoAbs as autoantigenic targets (centromere-associated proteins A, B, and C) [57, 76, 145–147].

As mentioned earlier, immunoblot was one of the first attempts to establish an appropriate multiplex test for the confirmation of ANA by using whole cell extracts with a similar autoantigen composition of HEp-2 cells [98, 148]. However, due to technical challenges, poor reproducibility, and loss of the native conformational structure of the relevant autoantigenic epitopes, this method was not established as a standard for multiplex autoAb analysis [19, 25, 149, 150].

As a result of improved purification methods for native autoantigens and progress in the expression of recombinant autoantigenic targets, the use of both molecule sources did not only enable the development of singleplex autoAb ELISAs but of multiplex dot or line immunoassays (D/LIAs), too [116, 140, 151]. In daily laboratory routine, D/LIAs have been established as one of the standard tests for ANA and ANCA confirmation [140, 152–154]. Moreover, D/LIAs appear to be an ideal solution for other serological diagnoses, where multiple autoAbs are required [155–158]. This holds not only true for CD serology where even a simultaneous IgA deficiency can be conducted apart from the (auto)Ab testing but proved to be very effective for the serology of SSc, DM/PM, or autoimmune liver diseases [140, 154]. Thus, D/LIAs with more than 20 autoantigenic targets have been introduced for the confirmatory diagnostics of ANA successfully [159]. Of note, the miniaturization of the technique by deploying sophisticated nanoliter dispensing devices and pattern recognition software for optical density reading render this technique most potential for future multiplex autoAb testing [160].

It should be noted in this context, that the attempts to employ the 96-well ELISA platform for autoAb multiplexing by using single wells for the immobilization of distinct antigens appear to be just an intermediate stage which was called into question very soon due to obvious shortcomings of the approach.

The progress in fluorescence reading as well as flow cytometry and microscopy paved the way for a new era in multiplexing [161–169]. Thus, several multiplex assay developments employing surface-activated microbeads coded by fluorescent dyes, size, or shape on the one hand and fluorescence microscopy or flow cytometry as read-out on the other hand were reported [170–172].

The intriguing biochip mosaic technology enabled multiplex autoAb IIF reading by using various cellular and tissue substrates on one solid phase [173–175]. Further, the luminex technology deploying fluorescence-coded microbeads and flow cytometry enabled the development of an intriguing and very successful multiplex autoAb detection technique [176, 177]. Very soon, this novel technology was commercialized by several companies. The possibility to detect several autoAbs and the high throughput led to the development of such multiplex autoAb systems like Athena and FIDIS or the fully automated BioPlex2000 system covering various serological autoimmune diagnoses [172, 177–179]. The growing success and the ready automation of the luminex technology were very appealing especially for larger laboratories with ever growing sample volumes [177]. Indeed, demand for autoAb testing started rising exponentially in the 1980s and this phenomenon called into question even the recommended two-tier strategy encompassing IIF as the ideal autoAb screening [180–182]. As a matter of fact, laboratories in particular in the USA have begun replacing IIF due to its major shortcomings, namely lack of automation, standardization, modern data processing, and experts in IIF reading [3, 43, 73, 170, 183, 184]. Although the newly developed luminex applications for autoAb testing helped to ease the pressure in terms of rising autoAb analyses, there was growing dissatisfaction among rheumatologists with the assay performance of the technology [42]. Indeed, false-negative ANA findings leading to ill-defined diagnoses raised the concern of clinicians [185, 186]. Consequently, the American College of Rheumatology (ACR) initiated a task force in 2009 investigating the issue [42]. In conclusion, IIF was confirmed as standard method for ANA reading and laboratories requested to return to the two-stage strategy or to make sure that clinicians requesting ANA testing are aware of the different assay performance by multiplexing [4].

Of note, despite the development of similar multiplex tests for ANCA testing, IIF was also not challenged as screening assay in the two-stage strategy yet.

## Improvement of IIF by Digital Fluorescence

The decision of the ANA task force of the ACR to retain the status of IIF and, thus, to confirm the two-stage strategy for CTD serology required an overhaul of the IIF technique badly [180, 181].

To employ this technique in a modern laboratory environment for CTD-associated antibody testing, the earlier mentioned shortcomings of IIF are needed to be addressed. In this context, the tremendous progress in fluorescence microscopy, image taking, and software development helped to usher in a new era of digital fluorescence [56, 161, 187, 188]. To the best of our knowledge, our group was the first to overcome critical disadvantages of ANA reading with IIF by introducing a standardized and automated fluorescence interpretation system which is based on the Videoscan technology and commercialized under the AKLIDES® brand [162, 189, 190]. AKLIDES® enables automated IIF reading by a sequential, multistage process including image acquisition by a CCD camera and software-controlled quality control, object segmentation, object description, and object classification by the use of novel pattern recognition algorithms. Thus, the system representing a composition of different hardware modules including a motorized inverse fluorescence microscope enables dynamic autofocusing resulting in the acquisition of quantitative fluorescence signals. The ensuing increased standardization and automation diminished the high intralaboratory and interlaboratory variability of ANA IIF reading, allowed the differentiation of cytoplasmic from nuclear staining, and rendered this method more applicable to high throughput screening [191–193].

Other diagnostic companies started developing similar systems and introducing new technologies for automated IIF pattern interpretation. In general, these commercially available systems are based on digital acquisition of fluorescence signals and most of them enable automated analysis of IIF images by pattern recognition algorithms (AKLIDES®, Medipan, Dahlewitz/Berlin, Germany; Nova View®, Inova, San Diego, USA; Zenit G Sight, A. Menarini Diagnostics, Grassina-Firenze, Italy; Europattern®, Euroimmun, Lübeck, Germany) [20, 194–196]. However, few systems distinguish between positive and negative screening results only (Helios, Aesku.Diagnostics, Wendelsheim, Germany; Image Navigator, Immuno Concepts, Sacramento, USA; Cytospot, Autoimmun Diagnostika, Straßberg, Germany) [185, 197]. In summary, all systems were reported to meet the demand for automated interpretation and satisfactory system performances were obtained by comparative studies at least for qualitative ANA evaluation [197, 198].

The fully automated interpretation system AKLIDES® was the first platform which performance was evaluated in clinical studies successfully [199–201]. Egerer et al. published the first clinical evaluation in 2010 by comparing the use of the new technology for ANA assessment of 1222 sera in the routine laboratory environment of both a university and a private referral laboratory [199]. An agreement of 93.0 % (859/924) and of 90.6 % (270/298) between automated AKLIDES® interpretation and classical ANA reading in the university and the private laboratory were reported, respectively. Remarkably, end-titer analysis based on quantitative fluorescence reading was shown for the first time, which overcomes a crucial shortcoming of IIF and levels it with other quantitative assay techniques established in routine clinical laboratories. Thus, the application range of the novel interpretation systems (AKLIDES®, Europattern®, NovaView®) was enlarged by adding ANCA and anti-dsDNA autoAb testing on human neutrophils and *Crithidia luciliae*, respectively [191, 202–204].

In summary, the intriguing development of these novel automated IIF interpretation systems strengthened the position of IIF as screening technique within the two-tier strategy for ANA and ANCA analyses. Thus, the demand of even large laboratories in terms of automated autoAb testing by IIF with modern data management could be addressed adequately. Tozzoli et al. concluded that a new technological era in the routine autoimmune laboratory was reached by the introduction of fully automated IIF in 2009 [180]. Furthermore, this technology may also stimulate clinical research regarding larger population studies, e.g., the prevalence of the dense-fine speckled (DFS) pattern, and hence, of the DFS70 autoAbs, in different apparently healthy and diseased populations [205].

## Combination of Screening and Confirmatory Testing

**Fig. 2:**
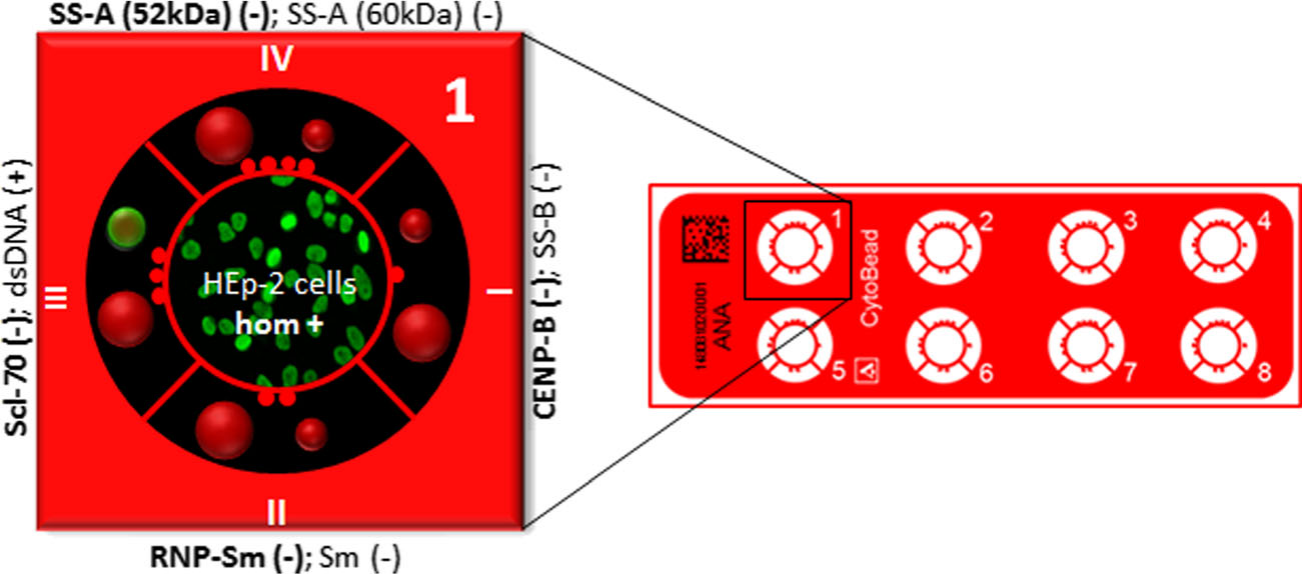
Multiplexing strategy of CytoBead® technology exemplified for CytoBead® ANA assay. Combination of ANA screening with HEp-2 cells (*middle part*) and anti-ENA testing with antigen-coated microbeads (*peripheral parts I–IV*) in one reaction environment. Example of an ANA positive serum with positive homogeneous fluorescence pattern on HEp-2 cells and positive signal on dsDNA-coated microbeads presented as green fluorescence halo (*small red microbeads in part III*). *ANA* antinuclear antibody, *CENP* centromere protein, *Da* Dalton, *dsDNA* double-stranded DNA, *ENA* extractable nuclear antigen, *hom* homogeneous, *RNP* ribonuclear protein, *Scl-70* DNA-Topoisomerase I, *Sm* Smith, *SS* Sjögren-Syndrome, (+) positive, (−) negative

Irrespectively of the tremendous progress in automated autoAb testing by IIF at the beginning of this millennium, the constraint to use two different assay techniques for the recommended two-stage strategy of ANA and ANCA analyses has not been abolished yet [4]. This strategy enables a plausibility control of the obtained results because specific autoAb assays may give false-positive findings. For instance, a positive anti-dsDNA finding in ELISA in combination with ANA negativity cannot be regarded as relevant regarding diagnosis of SLE. However, the possibility of false-negative findings using the two-tier strategy especially for ANA reading in terms of sera positive for autoAbs to SS antigen A (SS-A/Ro) is still eminent at hand and represents an essential drawback of such approach [206]. Only the combination of both stages in one multiplex test would overcome these shortcomings and provide an ideal solution for autoAb testing addressing key clinical and laboratory needs. As a fact, this intriguing idea is quite simple, and thus, it appears astonishing that no such attempt was undertaken earlier. Hence, combination of the advantages of cell-based assays and the potential for multiplexing by microbead immunoassay (MIA) employing IIF within one reaction environment could revolutionize autoimmune diagnostics (Fig. 2).

### Second-Generation ANA Testing

To realize the idea of combining autoAb screening and confirmation, we started developing a unique IIF reaction environment encompassing classical ANA analysis on HEp-2 cells and simultaneous multiplex detection of autoAbs by MIA. Indeed, merging screening and confirmatory testing for disease-specific autoAbs could generate many benefits including shorter hands-on times, better reproducibility of autoAb findings, and higher cost-effectiveness especially for larger sample series.

**Fig. 3:**
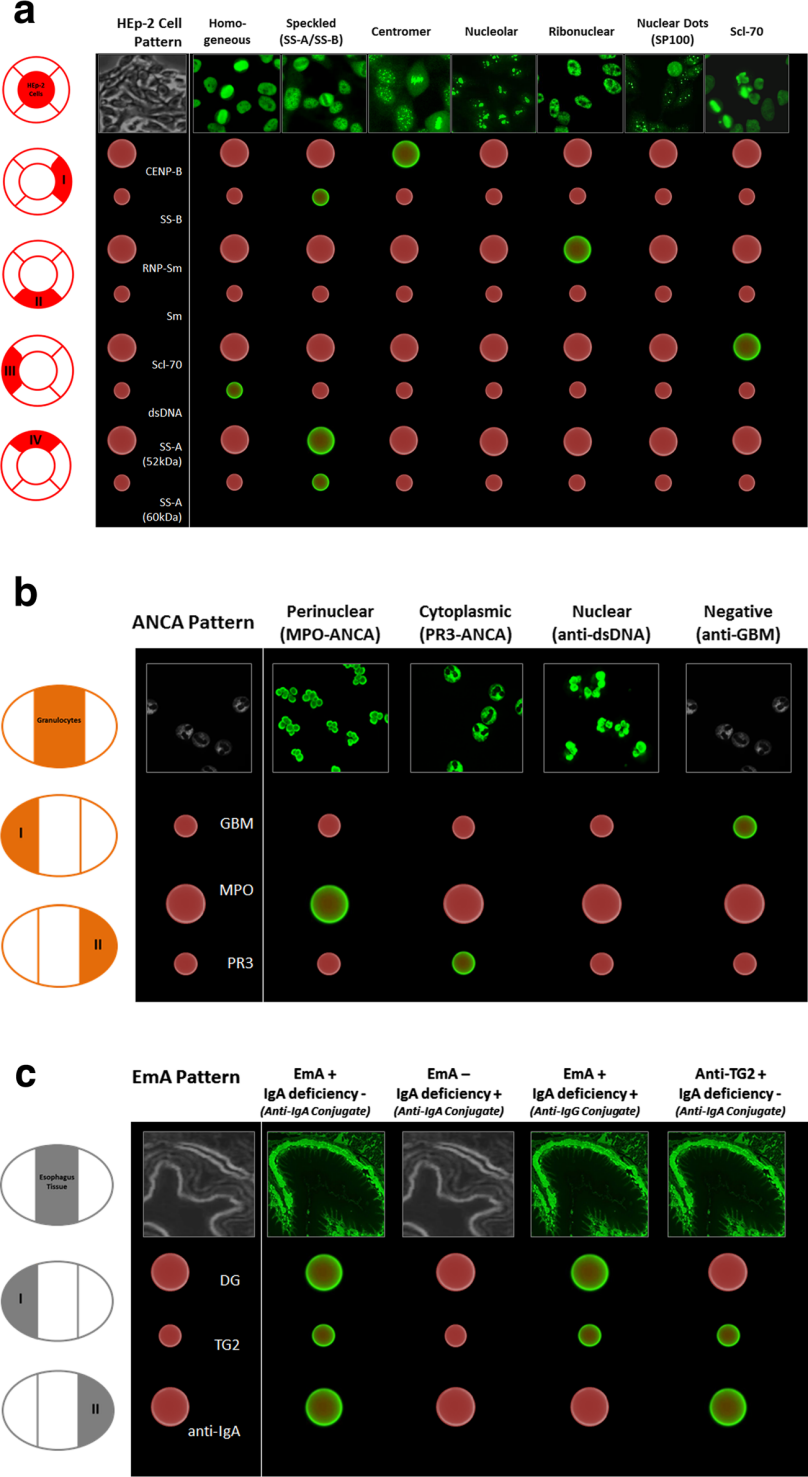
CytoBead® assays for the detection of **a** antinuclear antibodies (ANA) with CytoBead® ANA assay, **b** antineutrophil cytoplasmic autoantibodies (ANCA) with CytoBead® ANCA assay, and **c** celiac disease (CD)-specific (auto)antibodies (auto/Abs) with CytoBead® CeliAK assay. Matching principle of specific fluorescence patterns on HEp-2 cells (**a**), neutrophil granulocytes (**b**), and esophagus tissue (**c**) with positive reactions of antigen-coated microbeads immobilized in peripheral compartments. *CENP* centromere protein, *Da* Dalton, *dsDNA* double-stranded DNA, *EmA* endomysial antibody, *GBM* glomerular basement membrane, *MPO* myeloperoxidase, *PR3* proteinase 3, *RNP* ribonuclear protein, *Scl-70* DNA-Topoisomerase I, *Sm* Smith, *SS* Sjögren-Syndrome, (+) positive, (−) negative

First, a MIA which utilizes multiple carboxylated polymethylmethacrylate bead populations differing in size and/or concentrations of fluorescent dye for multiplexing was developed [207]. The classification of bead populations and measurement of corresponding ligand fluorescence intensity was readily performed by AKLIDES® enabling the detection of six different antinuclear autoAbs to Scl-70, Sm, SS-A (Ro60), SS-B (La), CENP-B, and, dsDNA. This assay development created the basis for the design of a unique IIF reaction environment which could integrate the classical ANA testing on HEp-2 cells in one test [102]. The new assay technique combining classical ANA testing with confirmatory analysis by MIA was termed CytoBead® technology (Fig. 3a). Intriguingly, the novel options of digital fluorescence enabling quantitative analysis not only of specific autoAb testing by MIA but also of classical ANA reading on HEp-2 cells can be readily employed by CytoBead® assays. Thus, they can be standardized by calibrated interpretation systems for automated autoAb testing. Consequently, this is a new age of standardization of ANA testing as a whole which was not feasible with classical ANA testing by IIF in the past.

Altogether, a new generation of autoAb testing could be established that can meet the demand of modern routine service laboratories for the serology of SARD/CTD by addressing the key disadvantages of the currently recommended two-stage autoAb testing.

Recently, this new assay referred to as second generation ANA testing was evaluated in a comprehensive serological study comprising inter alia 174 patients with SLE, 103 with SSc, 46 with SjS, 36 with RA, 13 with MCTD, 21 with DM/PM, 21 with infectious disease, 93 with autoimmune liver diseases, 78 with inflammatory bowel disease, and 101 blood donors [102]. The CytoBead® ANA simultaneously determines ANA on HEp-2 cells and autoAbs to dsDNA, CENP-B, SS-A/Ro52, SS-A/Ro60, SS-B/La, RNP-Sm, Sm, and Scl-70. The obtained good agreement of the CytoBead® ANA with classical ANA reading by IIF and ELISA supports the notion that the novel combined reaction IIF environment for one-step ANA analysis employing HEp-2 cells and autoantigen-coated fluorescent beads as respective targets can provide at least the same assay performance like classical two-tier ANA testing.

Furthermore, simultaneous detection of ANA and specific autoAbs such as to SS-A/Ro by CytoBead® ANA can almost eliminate the risk of false-negative findings and increase the already high negative predictive value of ANA testing. Of note, this is especially in the interest of rheumatologists who would like to exclude the presence of autoimmunity in their differential diagnosis of SARD by ordering ANA testing. In this study, 4/267 (1.5 %) ANA-negative patients with positive anti-SS-A or anti-CENP-B autoAbs were determined by second-generation ANA analysis. As a fact, these distinct patients with RA and SjS would have been missed by the currently recommended two-tier strategy since ANA negativity and positivity for anti-SS-A and anti-CENP-B autoAbs were confirmed by classical testing.

### New-Generation ANCA Testing

The CytoBead® technology was also applied for the comprehensive analysis of ANCA and the resulting CytoBead® ANCA was evaluated in terms of its assay performance [208]. Indeed, the combination of both IIF and antigen-specific assays was found in several studies to be the optimal strategy for ANCA detection and led to the recommendation of a two-stage ANCA testing.

Alike CytoBead® ANA development, after having designed a multiplex addressable MIA detecting MPO-ANCA, PR3-ANCA, and autoAbs against the noncollagen region of the alpha-3 subunit of collagen IV representing the glomerular basement membrane (GBM) antigen, a unique reaction environment for the additional detection of ANCA on fixed neutrophils was generated (Fig. 3b). The novel CytoBead® ANCA is a unique combination of a classical cell-based assay with multiplexing microbead technology [204, 208].

Sowa et al. recruited 592 patients including 118 patients with AAV, 133 with RA, 49 with infectious diseases, 77 with inflammatory bowel disease, 20 with autoimmune liver diseases, 70 with primary sclerosing cholangitis (PSC), and 125 blood donors and compared multiplex CytoBead® ANCA testing with classical methods such as IIF and ELISA [208]. Quantitative PR3- and MPO-ANCA analysis by multiplex CytoBead® technology turned out to be at least equal or better compared to classical ELISA testing for specific ANCA. Remarkably, automated endpoint ANCA titer analysis by only one serum dilution employing the automated interpretation system AKLIDES® revealed a very good agreement with the classical ANCA IIF on neutrophils. Another intriguing finding was the detection of PR3-ANCA in patients suffering from ulcerative colitis (UC) and PSC apart from those with GPA. These data appear to confirm a recent report of PR3-ANCA positive patients suffering from UC and PSC detected by another sensitive MIA technique [138]. Thus, the new reaction environment of the CytoBead® ANCA enables highly sensitive PR3-ANCA testing and might compete with third-generation ELISA in terms of assay performance.

Consequently, automated multiplex IIF combining screening and confirmatory ANCA testing in one test may replace the time-consuming current two-stage ANCA testing strategy by a one-step multiplexing CytoBead® analysis [206]. In context of the emergency diagnostics required for rapidly progressive glomerulonephritis, the novel multiplex ANCA analysis by CytoBead® appears to be an attractive approach to meet the clinical need for comprehensive ANCA testing in the fastest way possible.

### Comprehensive CD Serology

The serological diagnosis of CD comprises the detection of EMA and auto/Abs against deamidated gliadin and TG2 of the IgA isotype. As a fact, EmA detected by IIF is still considered the gold standard for (auto)Ab testing in CD [65]. To address the need for comprehensive CD-specific (auto)Ab testing in terms of workload and cost reduction in routine autoimmune laboratories, we developed a multiplex CytoBead® CeliAK assay (Fig. 3c) [209]. Multiplex CD-specific (auto)Ab testing might even be an attractive diagnostic tool in the context of the novel diagnostic criteria published by the European Society for Paediatric Gastroenterology, Hepatology and Nutrition (ESPGHAN) recently [65]. These criteria obviously strengthen the role of CD serology within the workup of patients with the suspicion of CD. Thus, CD can be diagnosed without histology by waiving duodenal biopsy in case of anti-TG2 autoAb IgA levels 10 times higher than the upper limit of normal (ULN) in patients positive for HLA-DQ2 or HLA-DQ8 and a positive response to gluten-free diet or confirmation by EmA testing.

Hence, the novel CytoBead® CeliAK was evaluated by investigating in total 380 patients and controls comprising 155 CD patients, 5 with IgA-deficiency, 68 with cystic fibrosis, 59 with eye diseases, and 93 blood donors [209]. Findings were compared with classical IgA-(auto)Ab analyses by ELISA and IIF. As a fact, the difference between CytoBead® and classical testing was only significant for anti-TG2 autoAb testing whereas the eight discrepant sera with anti-TG2 autoAb positivity by ELISA and negative levels by CytoBead® CeliAK belonged to four CD patients and four controls. Altogether, the CytoBead® CeliAK represents the first multiplex quantitative IgA anti-TG2 autoAb and anti-DG Ab multiplex assay which provides simultaneous EmA analysis as reference method and IgA deficiency testing. This comprehensive approach has the potential to improve CD serology and demonstrated excellent results regarding the great number of CD patients with anti-TG2 autoAb levels >10× ULN due to its high sensitivity. Additionally, due to the flexibility of the technique, further autoAbs such as those to GP2 stratifying CD patients further might be included [210, 211].

## Conclusion

Hitherto, the history of autoAb testing has been characterized by an intriguing development of several assay techniques to keep up with the tremendous progress in the understanding of autoimmune diseases and their appropriate diagnostics [180, 197]. Today, autoAb analysis is an integral part in the serological diagnosis of SARD like CTD and AAV and organ-specific autoimmune disorders [4, 26, 60]. Hence, there is no doubt that the introduction and further evolvement of IIF as one of the first autoAb-detecting assay techniques had and have an essential impact on this process [162, 197]. In the history of autoAb testing, various techniques emerged and were replaced by newer ones providing better assay performance and benefits regarding higher sample throughput and standardization [34, 80]. In this context, it is astonishing to note that IIF is still one of the key techniques to analyze autoAbs and even recommended as screening assay within the two-stage strategy for ANA and ANCA testing. In addition, IIF remains a reference method for the detection of distinct autoAbs like EmA in the serology of organ-specific autoimmune disorders indeed [65].

Despite the obvious benefits of IIF, this assay technique has been characterized by time consuming and subjective evaluation, insufficient automation, as well as poor standardization since its introduction [162]. In particular, pattern reading for ANA and ANCA testing was prone to inconsistencies in description and classification of respective staining patterns.

As a consequence, novel assay techniques based on solid-phase immunoassays like ELISA or multiplexing technologies creating the basis for different commercial platforms evolved and were introduced into routine autoimmune laboratories [149]. Nonetheless, IIF is still recommended to be used as the gold standard method for instance for ANA testing due to the unsatisfactory assay performance of even the latest multiplex technologies in this important area of autoAb analysis [4].

This situation changed dramatically by the development of digital fluorescence and its implementation in IIF testing. The breathtaking new options of pattern recognition combined with progress in automated fluorescence microscopy paved the way for the evolvement of an entirely new generation of automated interpretation systems [206]. Different commercially available IIF platforms for autoAb testing were designed and applied for ANA and ANCA reading in particular. First evaluation studies support the good performance of these systems and high agreement between visual and automated autoAb interpretation [212].

Of note, this enormous technology development comprising digital fluorescence image acquisition and automatic pattern recognition could be extended to other cell-based IIF assays in the search for new biomarkers. Thus, the quantification of γH2AX foci for DNA damage analysis, which used to be time consuming, subjective, and not suitable for high-throughput screening, could be standardized and automated [213, 214]. Successful evaluation studies support the introduction of this new DNA damage marker into clinical routine for cytostatic resistance development diagnostics [215].

Nevertheless, since the majority of clinical immunology laboratories follow the two-stage strategy for ANA and ANCA testing, substantial constraints regarding high-throughput and cost-effectiveness remain. The expansion of automated IIF interpretation systems like AKLIDES® to assess addressable MIAs created a unique novel assay platform allowing fully automated evaluation of cell-based screening tests and antigen-specific multiplex assays in one reaction environment for the first time. The evolvement of the CytoBead® technology combining quantitative autoAb screening and confirmatory testing in one IIF analysis enables second-generation autoAb detection in one test. This intriguing multiplex reaction environment addresses key needs for an effective standardized autoAb testing in laboratory routine. Major disadvantages of classical autoAb analysis by IIF were overcome by this new technique. First diagnostic applications for second-generation ANA and ANCA testing as well as comprehensive serology of CD-specific (auto)Abs were developed and successfully evaluated.
